# Paralyses: Cerebral, Bulbar, and Spinal. A Manual of Diagnosis for Students and Practitioners

**Published:** 1887-03

**Authors:** 


### Reviews of Books.

---

*Paralyses: Cerebral, Bulbar, and Spinal. A Manual of Diagnosis for Students and Practitioners.* By H. CHARLTON BASTIAN, M.A., M.D., F.R.S., Fellow of the Royal College of Physicians, &c., &c. With numerous Illustrations. London: H. K. LEWIS. 1886.

In a volume of considerable size, Dr. Charlton Bastian has provided a grammar of diagnosis, both pathological and regional, of the various forms of paralysis having their origin in the brain, bulb, and spinal cord. It is not pretended that all that is known about the several varieties of paralysis is fully stated in the work; but the author's endeavour has been to facilitate diagnosis by the student or practitioner, by setting forth so much concerning each form of paralysis as is necessary for its separate recognition. This, on the whole, is true, and a most useful and helpful work the one under consideration indeed is; at the same time, no one will be surprised if the author of *The Brain as an Organ of Mind* becomes occasionally a little discursive on some topics beyond the strict requirements of the diagnosing student, passing out of the realms of Medicine into those of Philosophy. For the most part, however, he considerately records these excursions in smaller print than the fundamental text, and even goes so far as to suggest (p. 101, *e.g.*) that the reader may skip them, if deemed supererogatory.

Of a work like the present, divided up into many chapters and sections, anything approaching an analytical review is out of the question here, and we can therefore but express an opinion upon it as a whole. The varieties of paralysis are now so numerous, and as yet, in some instances, so liable to be confused one with another, that a manual of differential diagnosis is a distinct desideratum; and few persons are better qualified than Dr. Bastian to compile such a book as this, which undoubtedly supplies a want. To two or three chapters we have turned with special and minute interest, in order to see what Dr. Bastian has to say upon the subjectmatter of them severally. Of most of these we would speak with unqualified satisfaction; but the chapter on the causation of contracture, ankle clonus, and exaggerated knee-jerk, as well as the chapter on paralyses due to lesions of the cerebellum, seem to be the neurology of the day before yesterday, not that of to-day. As to the existence of reflex paraplegia, Dr. Bastian is a sceptic; but obviously does not like to say as much unhesitatingly, for fear of wounding the beliefs of his surgical friends. The surgical medicine-man is a devout fetish-worshipper, and, "suckled in a creed out-worn" (neurologically speaking), often invokes the mythical deity, reflex paraplegia, in order to explain the, to him, inexplicable. His simple faith should be respected therefore even by the unbeliever, and not be ruthlessly destroyed by the iconoclastic physician! On p. 10 it is stated that peripheral paralysis will be dealt with only so far as it concerns the nerves that arise within the cranium. This is a mistake, when we know that many cases of paraplegia are not spinal in origin at all, but are affections of the peripheral distribution of spinal nerves, and demand efficient help, in order that they may be diagnosed from lesions of the spinal cord proper.

A word in conclusion about the illustrations. As the title-page says, they are numerous—numerically superior to their pictorial value. Most of them we have seen one or more times before, having advanced in decrepitude at each successive reproduction. Shade of Bewick! it is well that "mechanical" processes are coming more and more into vogue, to save thy art from its extremest profanation.

